# Health of 2-year-old children born after vitrified oocyte donation in comparison with peers born after fresh oocyte donation

**DOI:** 10.1093/hropen/hoab002

**Published:** 2021-02-19

**Authors:** Marjan Van Reckem, Christophe Blockeel, Maryse Bonduelle, Andrea Buysse, Mathieu Roelants, Greta Verheyen, Herman Tournaye, Frederik Hes, Florence Belva

**Affiliations:** 1 Faculty of Medicine and Pharmacy, Vrije Universiteit Brussel, Brussels, Belgium; 2 Centre for Reproductive Medicine, Universitair Ziekenhuis Brussel (UZ Brussel), 1090 Brussels, Belgium; 3 Department of Obstetrics and Gynaecology, School of Medicine, University of Zagreb, Zagreb 10000, Croatia; 4 Centre for Medical Genetics, Universitair Ziekenhuis Brussel (UZ Brussel), 1090 Brussels, Belgium; 5 Department of Public Health and Primary Care, Environment and Health/Youth Health Care, 3000 Leuven, Belgium; 6 Department of Obstetrics, Gynecology, Perinatology and Reproduction, Institute of Professional Education, Sechenov First Moscow State Medical University of the Ministry of Health of the Russian Federation (Sechenov University), Moscow 119992, Russia

**Keywords:** oocyte vitrification, oocyte donation, ICSI, health, offspring

## Abstract

**STUDY QUESTION:**

Does oocyte vitrification adversely affect the health of 2-year-old children compared with peers born after use of fresh oocytes in a donation programme?

**SUMMARY ANSWER:**

The growth and health of 2-year-old children born after oocyte vitrification are similar to those of peers born after use of fresh oocytes.

**WHAT IS KNOWN ALREADY:**

Although oocyte vitrification is a well-established procedure in ART, the evidence on its safety for offspring is limited. Currently, no disadvantageous effects of oocyte vitrification have been shown in terms of obstetric and neonatal outcome. However, no data beyond the neonatal period are available to date.

**STUDY DESIGN, SIZE, DURATION:**

A combined retrospective and prospective observational study was performed in a tertiary reproductive centre. The retrospective data were available in our extensive database of children born after ART. Donor cycles with an oocyte retrieval between January 2010 and March 2017 and a fresh embryo transfer resulting in the livebirth of a singleton were selected from the established oocyte donation programme. Fresh or vitrified oocytes were used in the donor cycles and all pregnancies in oocyte recipients were achieved after ICSI. Only children residing in Belgium were eligible for follow-up.

**PARTICIPANTS/MATERIALS, SETTING, METHODS:**

Biometric and health parameters of 72 children born after oocyte vitrification were compared with those of 41 children born after use of a fresh oocyte. Data were collected by means of questionnaires and physical examinations at the age of 21–30 months. The primary outcome measures were anthropometry and health at 2 years of age.

**MAIN RESULTS AND THE ROLE OF CHANCE:**

Length, weight, BMI, head circumference, left arm circumference and waist circumference at the age of 2 years were comparable between the vitrification and fresh group, also after adjustment for treatment, and maternal and neonatal characteristics (all *P* > 0.05). Health of the children in terms of hospital admission and surgical intervention rates were comparable between the vitrification and fresh group (both *P* > 0.05).

**LIMITATIONS, REASONS FOR CAUTION:**

Although the current study is the largest series describing health parameters beyond the neonatal period, the small numbers still preclude definite conclusions.

**WIDER IMPLICATIONS OF THE FINDINGS:**

This study provides the first evidence indicating that oocyte vitrification does not adversely affect the growth and health of offspring beyond the neonatal period.

**STUDY FUNDING/COMPETING INTEREST(S):**

This study was supported by Methusalem grants and by grants from Wetenschappelijk Fonds Willy Gepts, all issued by the Vrije Universiteit Brussel. All co-authors declared no conflict of interest in relation to this work. Both the Centre for Reproductive Medicine and the Centre for Medical Genetics from the UZ Brussel have received several educational grants from IBSA, Ferring, MSD and Merck for either research on oocyte vitrification or for establishing the database for follow-up research and organizing the data collection.

**WHAT DOES THIS MEAN FOR PATIENTS?**

Oocyte vitrification, a technique used for the deep-freezing of eggs, is a well-known procedure used in fertility clinics. However, vitrification might damage the eggs, which can negatively affect the fertility treatment or pose health risks for the offspring. Nevertheless, the information collected at birth so far has shown no differences between children born after use of frozen and non-frozen (or fresh) eggs. This study investigated the health of children at the age of 2 years, and found reassuring results. Indeed, this study shows that the health and growth of 2-year-old children born from a vitrified (frozen) egg is similar to that in children born from a fresh egg. This study is the first to provide evidence beyond infancy for the safety of using frozen eggs.

## Introduction

Oocyte vitrification, rather than slow freezing, is the preferred method for oocyte cryopreservation (Argyle *et al.*, 2016; De los Santos *et al.*, 2016; Rienzi *et al.*, 2017). Studies have shown that vitrified oocytes are equivalent to fresh oocytes in terms of fertilization, implantation, clinical pregnancy, ongoing pregnancy and live birth rates (Cobo *et al.*, 2008, 2010; Rienzi *et al.*, 2010, 2017; Cobo and Diaz 2011; Garcia *et al.*, 2011; Domingues *et al.*, 2017). Additionally, it has been demonstrated that oocyte vitrification does not negatively affect embryonic development, since no differences have been found between fresh and vitrified oocytes regarding cleavage rate (Cobo *et al.*, 2008; Garcia *et al.*, 2011), blastocyst formation rate (Garcia *et al.*, 2011) and embryo quality (Cobo *et al.*, 2008; Rienzi *et al.*, 2010).

However, as for many other types of ART, the technique was implemented without substantial evidence regarding safety in the offspring (Harper *et al.*, 2012). Therefore, guidelines recommend that more research on this topic must be performed (Practice Committees of American Society for Reproductive Medicine and Society for Assisted Reproductive Technology 2013). One of the main reasons for concern is that oocyte vitrification combines the use of high, possibly toxic, concentrations of cryoprotectants with ultra-rapid cooling rates, which can cause cryodamage to the oocytes (Cobo *et al.*, 2014; Konc *et al.*, 2014). Additionally, it is known that oocytes are particularly vulnerable to cryodamage (Smith and Silva 2004; Smith *et al.*, 2011; Konc *et al.*, 2014). Unfortunately, only a few studies investigating the safety of oocyte vitrification are available to date.

So far, the obstetric outcome after oocyte vitrification is reassuring. Pregnancy-related complications including antepartum haemorrhages, pregnancy-induced hypertensive disorders, gestational diabetes, cholestasis and preterm premature rupture of the membranes have been shown to be comparable between pregnancies conceived with vitrified and fresh oocytes (Cobo *et al.*, 2014). Furthermore, neonatal outcome is reassuring as well. Children born after oocyte vitrification have been shown to be comparable to peers in terms of mean birthweight and length, (very) low birthweight rate, mean gestational age, small for gestational age, mean APGAR score and neonatal hospitalisation rate (Chian *et al.*, 2008; Noyes *et al.*, 2009; Cobo *et al.*, 2014; Seshadri *et al.*, 2018). In addition, the rate of birth defects was comparable in a large series of 1027 children born after use of vitrified oocytes and 1224 born after use of fresh oocytes (adjusted odds ratio 0.81; 95% CI 0.53–1.20) even when the sample was restricted to donated oocytes (Cobo *et al.*, 2014), which is reassuring.

Although only a small number of studies is available, current evidence on the safety of oocyte vitrification indicates that vitrification does not have any additional obstetric or perinatal risk (Practice Committees of American Society for Reproductive Medicine and Society for Assisted Reproductive Technology 2013). However, no data beyond the neonatal period are available to date. In order to assess the health and development of 2-year-old children born after oocyte vitrification, the outcomes are compared with peers born after transfer of a fresh oocyte. The oocyte donation programme at the UZ Brussel provides a unique opportunity as it comprises the majority of all the oocyte vitrification procedures performed at our centre, as well as an appropriate parallel control group via fresh donation cycles. Indeed, since all study participants are donor recipients, confounding factors that potentially increase the obstetric risk in pregnancies conceived with heterologous oocytes (versus autologous oocytes) are waived. This study is part of the ongoing follow-up programme of ART children organized by the Centre for Medical Genetics of the UZ Brussel.

## Materials and methods

### Study participants

**Figure 1.**
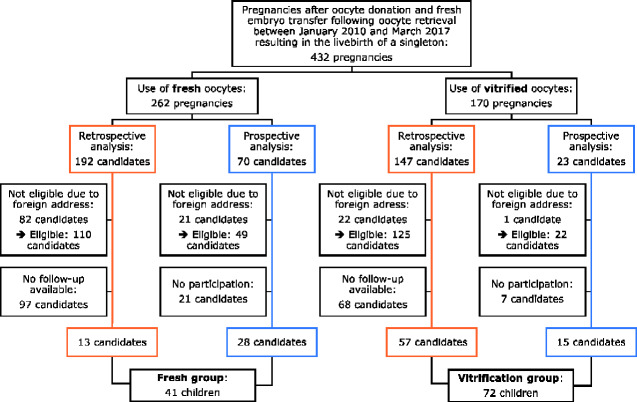
**Flowchart of the participants who were pregnant after use of fresh or vitrified donated oocytes**.

All donor cycles with an oocyte retrieval between January 2010 and March 2017 and a fresh embryo transfer resulting in the live birth of a singleton were selected. In total, 170 pregnancies after use of vitrified oocytes and 262 pregnancies after use of fresh oocytes met the criteria. The present study consisted of prospectively collected data supplemented with data available in our extensive database of children born after ART. Consequently, the results are based on two datasets (Fig. 1): one retrospective and one prospective, and include cycles with an oocyte retrieval from January 2010 to July 2015 and from August 2015 to March 2017, respectively. Given that outcome data are based on a physical examination performed in our centre, only children residing in Belgium were eligible for follow-up.

The retrospective dataset consists of 147 pregnancies in the vitrification group and 192 pregnancies in the fresh group. Of these candidates, 235 were eligible for inclusion. Required information for the aims of this study was available for 45.6% (57/125) of eligible candidates in the vitrification group and for 11.8% (13/110) of eligible candidates in the fresh group. The participation rate in the retrospective arm was 29.8% (70/235). Given this rather low participation rate, we approached both groups more actively and initiated a prospective cohort study.

The prospective part of the study comprises 23 candidates in the vitrification group and 70 candidates in the fresh group. Of these candidates, 71 were eligible for follow-up. In total, 60.6% (43/71) of these eligible candidates participated. Twenty-eight families from the prospective part (21 + 7) did not participate in the study for the following reasons: no interest in the study (n = 2), too busy to plan the hospital visit (n = 3), too far-removed from the hospital (n = 4), private reasons (n = 3), no-show on the planned appointment (n = 5) and 11 families could not be reached.

In short, 306 candidates were eligible for this study and a total of 113 participated: 72 (57 + 15) in the vitrification group and 41 (13 + 28) children in the fresh group.

### Oocyte donation programme

#### Laboratory procedures

The protocol for oocyte vitrification and warming has been described previously (De Munck *et al.*, 2016). Although its superiority over conventional IVF is not well established, in clinical practice ICSI is the preferred method for inseminating vitrified oocytes (Wood, 2012). In our study, all pregnancies in oocyte recipients were obtained after ICSI.

#### Recipient preparation

Patients received their fresh embryo transfer in an artificially prepared cycle; a standardized protocol for endometrial preparation was used, which we described earlier (Galvão *et al.*, 2019). With or without the use of GnRH agonist downregulation, oestrogens were administered orally using 2 mg oestradiol valerate (Progynova^®^, Bayer, Germany) twice daily for 6 days from day 3 of the menstrual cycle, increased to three times a day for another 7 days. A scan and blood sampling were carried out on day 13. If the endometrium was adequately primed (see above), a daily vaginal administration of 600 mg micronized progesterone (Utrogestan^®^, Besins, Belgium) was administered. Cleavage stage embryo transfer was planned on the fifth day of progesterone supplementation, and blastocyst transfer on the seventh day of progesterone administration.

### Study protocol

A comprehensive follow-up programme for children born after ART has been set up in our centre since the introduction of IVF in clinical practice and has been constant over the years (Bonduelle *et al.*, 2002). It consists of the combination of questionnaire data and results from physical examinations. Characteristics of the fertility treatment are obtained from the medical records of the hospital. The following indications for oocyte donation were taken into account: advanced maternal age (i.e. ≥40 years), premature ovarian failure (POF), poor responders to ART treatment (i.e. women with poor oocyte number and/or embryo quality or multiple previous failed attempts to conceive via ART), genetic conditions (i.e. conditions with and without direct influence on fertility), mutual donation (motherhood sharing) within a lesbian couple, endometriosis, poor embryo quality, a tubal factor for infertility and an additional male factor for infertility.

After the expected delivery date, parents are sent a questionnaire. Demographic data are obtained from the parents and obstetric and neonatal data are obtained from the gynaecologist and/or paediatrician. In case of a live birth and a residency in Belgium, the parents are invited for a detailed morphological assessment of their child(ren) at the Medical Genetics outpatient clinic, which is run by certified paediatricians. At the time of the visit, the questionnaire is verified with the parents and completed when necessary.

A second visit of the child takes place at ±2 years of age and is performed by a paediatrician, who is blinded to the mode of conception. Information regarding postnatal illnesses, hospital admissions, surgical interventions and medication intake is gathered and added to the files. The clinical examination at the age of 2 years included the measurement of weight, length, head circumference, left mid-upper arm circumference and waist circumference, and an assessment of major and minor malformations.

### Definitions

A major malformation was defined as a malformation causing functional impairment and/or requiring surgical intervention. All other malformations were classified as minor and were documented in accordance to a checklist based on the textbook by Aasse (1990; Bonduelle *et al.*, 2002).

Pregnancy-induced hypertension and pre-eclampsia were defined according to guidelines defined by the American College of Obstetricians and Gynaecologists (2013) as blood pressure elevation after 20 weeks of gestation in the absence of proteinuria or systemic findings and as (*de novo*) hypertension (diastolic blood pressure of ≥90 mm Hg) and (substantial) proteinuria (≥300 mg in 24 h) at or after 20 weeks of gestation, respectively.

### Ethics committee

The study was approved by the ethical committee of the UZ Brussel (B.U.N. 143201837044).

### Statistical analysis

Statistical analysis was performed using SPSS version 26.0 for Windows (IBM, USA). Categorical variables are presented as a count and percentage (%) and continuous variables as the mean and SD. Between-group differences were analysed using a chi-squared test for categorical variables and an independent Student’s *t*-test test for continuous variables. Statistical significance was set at the 5% level (*P*-value <0.05). The current sample sizes allow us to obtain statistical significance when groups differ by approximately more than 6–10% for dichotomous variables, and more than 0.4 times the SD for continuous variables. Anthropometric outcomes at birth are expressed as standard deviation scores (SDS), to correct for the gestational age and gender according to the growth charts constructed by Niklasson and Albertsson-Wikland (2008). Anthropometric outcomes at 2 years of age are expressed as SDS according to the Belgian growth reference (Roelants *et al.*, 2009).

Differences in birth parameters and anthropometric outcomes at the age of 2 years between the groups (vitrification or fresh) were additionally compared with multiple linear regression analysis adjusted for relevant covariates.

Preliminary univariate regression analysis was performed in order to select covariates, known to affect body size at birth and/or to be statistically different among the two study groups, for inclusion in the final models. The following covariates were tested: treatment variables (cleavage stage embryo transfer, single embryo transfer, indication for oocyte donation) and maternal characteristics (BMI, nulliparity, ethnic origin, pregnancy-induced hypertensive disorder) (see results section). Oocyte indication was stratified into five categories: advanced maternal age (≥40 years), POF, genetic condition, female factor (poor responders, bad embryo quality, endometriosis, tubal factor), non-female factor (same-sex lesbian couple, male factor).

For reasons of uniformity, the same covariates were used in the linear regression model at birth and at 2 years of age. Outcomes at 2 years were additionally adjusted for neonatal characteristics (birth weight SDS).

Results are expressed as unstandardized regression coefficients (B) with a 95% CI. This provides an estimate of the mean difference in SDS variables between the vitrification and the fresh group.

The association between the duration of time that an oocyte was vitrified and birth weight SDS was analysed with linear regression.

## Results

### Characteristics of the study population

In both groups, the majority of the embryo transfers were performed at the cleavage stage (97.2% and 70.7% in the vitrification and fresh group respectively; *P* < 0.001) (Table I). The proportion of single embryo transfers was comparable between the two groups (*P* = 0.41). In 2.8% of the vitrified-oocyte cycles, donor sperm was used compared with 34.1% of the fresh-oocyte cycles (*P* < 0.001). Fresh semen was used in 93.1% of the vitrified-oocyte cycles and in 63.4% of the fresh-oocyte cycles (*P* < 0.001). The age and BMI of both the father and mother were comparable between the vitrification and fresh group (all *P* > 0.05). In the vitrification group, 13 mothers (18.0%) were of non-Caucasian origin (11 non-Hispanic black, two Asian) while in the fresh group five mothers (12.2%) were of non-Caucasian origin (one Hispanic, three non-Hispanic black, one Asian) (*P* = 0.59). A comparable number of mothers were nulliparous: 75.8% in the vitrification group and 76.5% in the fresh group and (*P* = 0.93).

**Table I:** Treatment, parental and pregnancy characteristics.

	Vitrification	Fresh	*P*-value
**Treatment characteristics**			
Cleavage stage embryo transfer	70 (97.2%)	29 (70.7%)	<0.001
Single embryo transfer	22 (30.5%)	16 (39.0%)	0.41
Donated semen	2 (2.8%)	14 (34.1%)	<0.001
Fresh semen	67 (93.1%)	26 (63.4%)	<0.001
**Parental characteristics**			
Age father at birth (years) (n = 97)	40.6 (6.5)	40.9 (6.4)	0.82
BMI father (kg/m²) (n = 90)	25.7 (3.7)	26.2 (3.2)	0.55
Donor age at oocyte retrieval (years) (n = 106)	29.2 (3.8)	29.7 (5.0)	0.62
Age mother at birth (years) (n = 113)	39.3 (5.0)	38.6 (5.0)	0.46
BMI mother (kg/m²) (n = 109)	24.7 (4.9)	24.4 (4.2)	0.75
Maternal Caucasian race (n = 113)	59 (82.0%)	36 (87.8%)	0.59
Nulliparity (n = 100)	50 (75.8%)	26 (76.5%)	0.93
**Course of pregnancy**			
Weight gain during pregnancy (kg) (n = 109)	11.9 (4.6)	10.7 (3.9)	0.20
Maternal smoking during pregnancy (n = 111)	2 (2.8%)	0 (0.0%)	0.18
Maternal alcohol consumption during pregnancy (n = 112)	2 (2.8%)	2 (4.9%)	0.58
Medication intake during pregnancy (n = 113)	45 (62.5%)	24 (58.5%)	0.68
Pre-existing disease (n = 113)			
Thyroid disorder	8 (11.1%)	5 (12.2%)	0.86
Hypertension	1 (1.4%)	1 (2.4%)	0.69
Allergy and Asthma	3 (4.2%)	4 (9.8%)	0.25

Continuous data are expressed as mean ± SD and categorical data as number (%).

Categorical variables were compared with a chi-squared test and continuous variables with an independent samples Student’s *t*-test.

Regarding the course of the pregnancy, there was no difference between groups in weight gain of the mothers during pregnancy (*P* = 0.2) (Table I). There was also no difference regarding the intake of medication and substance abuse (smoking, alcohol) during pregnancy (all *P* > 0.05). Comparable proportions of mothers in both groups had pre-existing diseases (all *P* > 0.05).

The family constitution differed between the two groups: there was one single mother (1.4%) in the vitrification group and four (9.8%) in the fresh group (*P* < 0.001); none of the children in the vitrification group had homosexual parents while this was the case for one-fifth of the children in the fresh group (22%).

The mean duration of time that an oocyte was vitrified before warming was 149 days (range: 2–738 days).

The indications for use of donated oocytes for all 113 recipients are presented in Table II. Most recipients had more than one indication and therefore appear multiple times. The major indications for oocyte donation in the study population were advanced maternal age, poor response to ovarian stimulation and POF. The cause of POF was mainly idiopathic except for two women in the vitrification group with POF caused by ovariectomy and one woman in the fresh group with POF after chemotherapy. Genetic indications included conditions affecting fertility, such as Turner syndrome and carriers of fragile X pre-mutations, as well as other conditions with a more complex effect on fertility, for example translocations and inheritable diseases.

**Table II:** Indications for use of donated oocytes.

	Vitrification	Fresh	*P*-value
***Indication*^a^**			
Advanced maternal age	39 (54.2%)	21 (51.2%)	0.84
POF^b^	34 (47.2%)	7 (17.1%)	0.002
Genetic conditions	14 (19.4%)	5 (12.2%)	0.43
Turner syndrome	1	0	
Fragile-X premutation	3	1	
Other	10	4	
Poor responders	31 (43.1%)	17 (41.5%)	1.00
Poor embryo quality	10 (13.9%)	9 (22.0%)	0.30
Endometriosis	11 (15.3%)	8 (19.5%)	0.60
Tubal factor	7 (9.7%)	3 (7.3%)	0.74
Same-sex lesbian couple	0 (0.0%)	9 (22.0%)	<0.001
Additional male factor	18 (25.0%)	6 (14.6%)	0.24
***Number of indications for use of donated oocytes***			
Single indication	16/72 (22.2%)	14/41 (34.2%)	0.19
Multiple indications	56/72 (77.8%)	27/41 (65.8%)	

^a^ Couples with multiple indications will appear more than once.

^b^ POF (premature ovarian failure) excluding associated genetic conditions like Turner Syndrome and Fragile-X premutation.

Results are expressed as number (%). A chi-squared test was used to compare both groups.

To ensure that the studied population was not subject to selection bias, a non-participation analysis was performed to compare participants (n = 113) with non-participants (n = 193). The parameters compared included treatment variables (day of embryo transfer, donor age), paternal (BMI) and maternal (age, BMI, parity, pregnancy-induced hypertension, gestational diabetes) characteristics and neonatal outcome measures (gestational age, birthweight, birth length, head circumference). In the vitrification group, comparable results were found between participants (n = 72) and non-participants (n = 75) for all parameters (all *P* > 0.05). In the fresh group, a day-3 embryo transfer was performed in a higher proportion of the non-participants (n = 118) compared to the participants (n = 41) (85.4% versus 70.7%; *P* = 0.02). Nevertheless, the majority of the embryo transfers was performed at the cleavage stage in both groups. No other differences were found between participants and non-participants in the fresh group (all *P* > 0.05). Furthermore, when comparing all participants with non-participants, regardless of the use of fresh or vitrified oocytes, no differences were found concerning the investigated parameters (all *P* > 0.05).

### Obstetric outcome

Obstetric outcomes and complications are presented in Table III. The incidence of hyperemesis requiring hospitalisation was significantly higher in pregnancies conceived with fresh donated oocytes (4.9%) compared with vitrified donated oocytes (0.0%; *P* = 0.04). Pregnancies from the vitrification group were significantly more often complicated by pregnancy-induced hypertensive disorders (23.6%) compared with the fresh group (7.3%; *P* = 0.04). No differences were found in the occurrence of pre-eclampsia (*P* = 0.32) or haemolysis, elevated liver enzymes and low platelet count syndrome (HELLP)-syndrome (*P* = 0.55) between the groups. The incidences of other obstetrical outcomes were comparable between groups (all *P* > 0.05).

**Table III:** Obstetric outcomes and complications.

	Vitrification (n = 72)	Fresh (n = 41)	*P*-value
Vaginal bleeding			
<20 weeks	19 (26.4%)	11 (26.8%)	0.96
>20 weeks	1 (1.4%)	1 (2.4%)	0.69
Hyperemesis requiring hospital admission	0 (0.0%)	2 (4.9%)	0.04
Hypertensive disorders	17 (23.6%)	3 (7.3%)	0.04
Pregnancy-induced hypertension	6 (8.3%)	1 (2.4%)	0.41
Pre-eclampsia	8 (12.5%)	2 (7.3%)	0.32
HELLP syndrome	3 (4.2%)	0 (0.0%)	0.55
Abnormal placentation			
Placenta praevia	1 (1.4%)	0 (0.0%)	0.34
Placenta abruption	0 (0.0%)	1 (2.4%)	0.15
Preterm labour and PROM	5 (6.9%)	2 (4.9%)	0.66
Gestational diabetes	10 (13.9%)	5 (12.2%)	0.80
Cholestasis	3 (4.3%)	0 (0.0%)	0.10
IUGR	2 (2.8%)	0 (0.0%)	0.18

Results are expressed as number of cases (%). A chi-squared test was used to compare both groups.

PROM, premature rupture of the membranes; HELLP syndrome, haemolysis, elevated liver enzymes and low platelet count syndrome; IUGR, intra-uterine growth restriction.

The mode of delivery was comparable between groups, with 55.6% of children in the vitrification group and 53.6% of children in the fresh group being delivered by caesarean section.

### Results of univariate linear regression analysis

All treatment variables were tested and found not to affect birthweight SDS, that is cleavage stage embryo transfer, single embryo transfer, indication for oocyte donation (all *P* > 0.05). The variables maternal BMI, nulliparity and ethnic origin were tested and were not found to affect birthweight SDS (all *P* > 0.05). However, pregnancy-induced hypertensive disorder was found to affect birthweight SDS: there was a trend to a lower birthweight SDS in children whose mother had a pregnancy-induced hypertensive disorder (B: −0.4; 95% CI: −0.8; 0.0; *P* = 0.05).

For the final models, the covariate pregnancy-induced hypertensive disorder as well as the variables that differed between groups (cleavage stage embryo transfer, indication for oocyte donation) were used in the multiple linear regression analysis of neonatal outcomes and outcomes at 2 years of age.

### Neonatal outcome

The neonatal outcome is presented in Table IV. Mean gestational age and prematurity rate were comparable between the groups (both *P* > 0.05). Babies from the vitrification group had a slightly lower mean birthweight (3200 versus 3404 grams) and birthweight SDS (−0.18 versus 0.24) compared with babies from the fresh group (*P* = 0.04 and *P* = 0.01 respectively).

**Table IV:** Neonatal outcomes.

	Vitrification	Fresh	*P*-value	Adjusted^a^ mean difference, 95% CI
Gestational age (weeks) (n = 113)	38.9 (2.0)	38.9 (1.6)	0.97	
Premature birth <37 weeks (n = 113)	8 (11.1%)	3 (7.3%)	0.50	
Male sex (n = 113)	34 (47.2%)	22 (53.7%)	0.51	
Birthweight (g) (n = 113)	3200 (515.28)	3404 (474.03)	0.04	
SDS	−0.18 (0.84)	0.24 (0.86)	0.01	−0.3 (−0.7; 0.1)
Birth length (cm) (n = 113)	49.9 (2.6)	50.3 (2.3)	0.40	
SDS	0.23 (1.03)	0.37 (1.12)	0.52	−0.1 (−0.6; 0.4)
Head circumference at birth (cm) (n = 95)	34.7 (1.5)	34.3 (1.5)	0.22	
SDS	0.36 (0.93)	0.04 (1.02)	0.14	0.4 (−0.1; 0.9)
APGAR (n = 95)				
1min <4	3 (4.9%)	0 (0.0%)	0.10	
1 min <7	9 (14.8%)	4 (11.8%)	0.68	
5 min <4	0 (0.0%)	0 (0.0%)	NA	
5 min <7	5 (8.2%)	2 (5.9%)	0.67	
Neonatal admission (n = 113)				
Any	18 (25.0%)	5 (12.2%)	0.10	
≥7 days	10 (13.9%)	1 (2.4%)	0.15	

SDS, standard deviation score.

Categorical variables are presented as number of cases (%) and continuous variables as mean (SD) or mean difference (95% CI) from the reference group.

Categorical variables were compared with a chi-squared test and continuous variables with an independent samples Student’s *t*-test or ^a^multiple linear regression adjusted for covariates (day of embryo transfer, indication for oocyte donation, pregnancy-induced hypertensive disorder).

However, this difference was not statistically significantly different after adjustment for treatment and maternal characteristics (Table IV). Other anthropometrical parameters at birth were comparable between groups, even after adjustment for covariates (all *P* > 0.05). APGAR scores and neonatal admission rates were also comparable between groups.

The duration of time that an oocyte was vitrified was not associated with birthweight SDS (*P* = 0.45).

### Health outcome, including anthropometrics, at 2 years

The mean age of the children at follow-up was approximately 2 years and 2 months. The anthropometrical parameters of the children at the time of evaluation are presented in Table V. Length, weight, BMI, head circumference, left arm circumference and waist circumference at the age of 2 years were comparable between the vitrification and fresh group (all *P* > 0.05). Furthermore, adjustment for treatment, maternal and neonatal characteristics did not change this outcome (Table V).

**Table V:** Anthropometry at 2 years of age.

	Vitrification	Fresh	*P*-value	Adjusted^a^ mean difference, 95% CI
Age at evaluation (years) (n = 113)	2.21 (0.17)	2.17 (0.18)	0.26	
Weight (kg) (n = 111)	12.9 (1.5)	12.9 (1.3)	0.94	
SDS	−0.02 (1.04)	0.01 (0.98)	0.89	0.2 (−0.2; 0.6)
Length (cm) (n = 109)	89.8 (3.9)	89.9 (3.7)	0.90	
SDS	0.28 (1.06)	0.39 (1.13)	0.63	0.0 (−0.4; 0.5)
Head circumference (cm) (n = 110)	49.8 (4.7)	49.2 (1.4)	0.39	
SDS	0.71 (3.39)	0.20 (0.92)	0.36	0.8 (−0.5; 2.1)
Left arm circumference (cm) (n = 98)	16.1 (1.2)	16.2 (0.9)	0.58	
SDS	0.08 (0.95)	0.16 (0.68)	0.64	0.0 (−0.4; 0.4)
Waist circumference (cm) (n = 96)	49.4 (4.2)	50.8 (4.0)	0.12	
SDS	0.63 (1.23)	1.01 (1.13)	0.13	−0.2 (−0.8; 0.3)
BMI (kg/m²) (n = 109)	16.0 (1.36)	16.0 (1.13)	0.92	
SDS	−0.25 (1.13)	−0.22 (0.95)	0.90	0.1 (−0.3; 0.6)

Continuous variables are presented as mean (SD), or mean difference (95% CI) from the reference group.

Continuous variables were compared with an independent samples Student’s *t*-test or ^a^multiple linear regression adjusted for covariates (birthweight SDS, day of embryo transfer, indication for oocyte donation, pregnancy-induced hypertensive disorder).

There was no difference between the vitrification and fresh group in the occurrence of chronic illness other than recurrent minor infections (4.2% versus 7.3%, respectively). One child in the vitrification group suffered from a neonatal ischaemic cerebrovascular accident with convulsions, for which it received anti-epileptic medication; in the fresh group no medication intake was noted (1.4% versus 0%, respectively).

A comparable number of children was hospitalised during their infancy (vitrification group: 19.4%; fresh group: 24.4%). The main indication for hospitalisation was infectious disease for which 13 children in the vitrification group (18.1%) and eight children in the fresh group (19.5%) were admitted.

A surgical intervention was performed in five children from the vitrification group (6.9%) and seven children from the fresh group (17.1%) (*P* = 0.1). One child from the vitrification group and two children from the fresh group underwent two interventions.

In both groups, two children had a major malformation (vitrification group: 2.8%; fresh group: 4.8%). The observed major malformations were a hip dislocation and luxation and a polymalformative syndrome in the vitrification group, and a pyloric stenosis and a unilateral cryptorchidism in the fresh group. Minor malformations were found in four children in the vitrification group (5.6%) and in six children from the fresh group (14.6%).

## Discussion

To the best of our knowledge, this is the first study reporting on the health outcome of children born after oocyte vitrification beyond the neonatal period. We found that the anthropometrical parameters of children at the age of 2 years were comparable between the vitrification and the fresh group, even after adjustment for treatment, maternal and neonatal characteristics. Furthermore, health during childhood was comparable between the groups: a similar number of children suffered from chronic illness, were hospitalised for medical indications or had surgery performed. We also reported major and minor malformations, but the results should be interpreted with caution because of the limited numbers.

By extension, our study indicates that oocyte vitrification does not adversely affect the medical development of its offspring and that it accordingly can be considered a safe ART procedure, at least up to the age of 2 years. Although there are currently no other studies on the health of 2-year olds born after oocyte vitrification, our results are in line with studies describing 2-year olds born after embryo vitrification showing comparable motor and mental development between these children and peers born after spontaneous conception or a fresh embryo transfer (Sutcliffe *et al.*, 1995; Nakajo *et al.*, 2004).

Noteworthy in this study is that one-fifth of the children in the fresh group had homosexual parents in contrast with none of the children in the vitrification group. This difference can be explained by the preference of a treatment protocol using fresh oocytes for non-anonymous donations, such a mutual donations between lesbian couples, at our centre. Further, we observed that the use of donated, as well as frozen, semen samples was higher in the fresh group. This can be attributed to the higher proportion of oocyte donations between lesbian couples in the fresh group using donated, thus frozen semen samples, but whether this increased use of donor sperm had an impact on the outcomes for the offspring is beyond the scope of the study. Furthermore, we observed a significant proportional difference in timing of embryo transfer between groups. Nevertheless, in the majority of all cycles, embryos were transferred at the cleavage stage, making the clinical impact of this difference less likely.

Pregnancies from the vitrification group were significantly more complicated by pregnancy-induced hypertensive disorders compared with the fresh group. Although only few studies are available in literature, equivalent obstetrical outcomes have been reported between the use of vitrified and fresh oocytes (Cobo *et al.*, 2014). However, contrary to the study of Cobo *et al.* (2014), our study included exclusively pregnancies after heterologous oocytes, which might add to the discordant findings, since an adverse obstetric outcome has been repeatedly reported in donor oocyte pregnancies (Klatsky *et al.*, 2010; Stoop *et al.*, 2012; Blazquez *et al.*, 2016; Storgaard *et al.*, 2017; Moreno-Sepulveda and Checa 2019). In addition, the fact that 15% of the mothers in the vitrification group (versus 7% in the fresh group) were from non-Hispanic black origin might also have contributed to the finding of a higher rate of pregnancy-induced hypertensive disorders, which have been reported to be linked to black ethnicity (Grobman *et al.*, 2018).

Hypertensive disorders have been described as a common obstetric complication after embryo vitrification (Wikland *et al.*, 2010; Barsky *et al.*, 2016; Sites *et al.*, 2017; Roque *et al.*, 2018). There are two hypotheses regarding the pathophysiological processes leading to this adverse outcome. First, the vitrification process itself may affect the embryo. It is possible that vitrification alters epigenetic and/or metabolic processes in the pre-implantation embryo, contributing to an unfavourable obstetric and neonatal outcome (De Munck *et al.*, 2015; De Munck and Vajta 2017; Van Heertum and Weinerman 2018; Dall’Agnol and García Velasco 2020). Also, researchers suggested that vitrification might damage the trophectoderm cells of the blastocyst leading to aberrant placentation and subsequent negative pregnancy outcomes (Wikland *et al.*, 2010; Barsky *et al.*, 2016). Accordingly, it may be possible that vitrification damages oocytes as well, impairing their developmental potential, and also resulting in adverse outcomes. The underlying mechanisms have yet to be elucidated. The second hypothesis suggests the involvement of the hormonal environment during early pregnancy. For example, the difference in endometrial receptivity between fresh and frozen embryo transfer cycles, caused by the variation in treatment protocols (artificial cycles using hormonal replacement therapy versus (modified) natural cycles), may contribute to the discrepancy in obstetric outcomes (Johnson *et al.*, 2019; Roque *et al.*, 2019; Dall’Agnol and García Velasco 2020). Also, recent evidence shows an increased risk of hypertensive disorders in the absence of a corpus luteum (von Versen-Höynck *et al.*, 2019). It is clear that more research is necessary to elucidate the possible causal factors increasing the obstetric risk in pregnancies conceived with vitrified embryos/oocytes, particularly in a oocyte donation programme. Recently, Blazquez *et al.* (2018) suggested a minor role of cryodamage, since their study showed no differences in hypertensive disorders between fresh and vitrified-warmed embryos derived from fresh donated oocytes when the endometrial preparation and hormonal state is comparable in the recipients. Whether this also accounts for fresh and vitrified-warmed donated oocytes needs to be confirmed in larger studies.

In our study, babies from the vitrification group had a lower mean birthweight (3200 versus 3404 grams, respectively; *P* = 0.04) and a lower birthweight SDS (−0.18 versus 0.24, respectively; *P* = 0.01) compared to babies from the fresh group. This can be explained by the higher proportion of pregnancies complicated by hypertensive disorders observed in the vitrification group. It is well known that maternal hypertensive conditions during pregnancy are associated with a higher risk of adverse effect on the offspring, including a lower mean birthweight (Browne *et al.*, 2015; Phad *et al.*, 2015). While the effect of this pregnancy-induced maternal hypertensive condition on the birthweight of offspring was only marginal in our study with modest sample size, the difference in birthweight between the vitrification and the fresh group was no longer statistically significant in the adjusted analysis. Consequently, our findings correspond to literature reports indicating that oocyte vitrification does not adversely impact the neonatal outcome of offspring (Chian *et al.*, 2008; Noyes *et al.*, 2009; Cobo *et al.*, 2014; De Munck *et al.*, 2016; Seshadri *et al.*, 2018).

The strengths of this study are that it is a single-centre study using standardised protocols and that the study population comprised pregnancies from donated oocytes only. The fact that all pregnancies resulted from fresh embryo transfers in an artificially prepared cycle adds to the homogeneity of the study groups and the generalisability of the results. Furthermore, the current data, which are the largest in the literature describing the health of young children born after oocyte vitrification, are obtained from a thorough medical examination, which is part of our follow-up programme of ART children, ongoing for over more than 20 years.

Some limitations of this study should be mentioned. For instance, even though we present data from the largest cohort of 2-year olds to date, the number of children is still too low to draw definite conclusions, particularly regarding congenital malformations. Furthermore, although the participation rate seems rather low, 60.6% of the approached families participated in the prospective arm of this study, with comparable proportions in the fresh and vitrification groups. Also, the children were enrolled using the same strategy in both the vitrification and fresh group, thus avoiding a possible recruitment bias. Unfortunately, a much lower participation rate of 29.8% was reached in the retrospective part of the study. In particular, the low number of participating candidates in the fresh group (11.8%), as opposed to the vitrification group (45.6%), contributes to the low overall rate in the retrospective data. This low participation rate in the fresh group can be explained by the fact that children born after use of fresh oocytes were initially randomised for follow-up at our centre whereas children born after use of vitrified oocytes were not. This means that, unlike the children born from vitrified oocytes, only one in three children born from fresh oocytes were randomly selected to be invited for follow-up at our centre, an approach applied because of the large number of children conceived in our centre and the lack of capacity to include all for research. However, owing to the discrepancy in participation rates between the two groups, a prospective approach was initiated. Furthermore, the strict selection of only those participants with complete and qualitative data for this study may contribute to the modest participation rate as well. An inherent risk of non-participation is the introduction of a selection bias leading to non-representative results. Therefore, we compared several treatment, parental and neonatal characteristics of participants and non-participants from both the fresh and vitrification group. The analysis showed no differences between the participants and non-participants in the vitrification group. In the fresh oocyte group, however, the proportion of cleavage-stage embryo transfers was higher in the non-participants as opposed to the participants. Still, the majority of the fresh embryo transfers was performed at the cleavage stage in both participants and non-participants. Therefore, this difference probably has no clinically significant influence on our outcomes. Nevertheless, our results must be interpreted with caution.

In conclusion, this study shows that the growth and health of 2-year-old children born after oocyte vitrification is comparable to those of peers born after use of fresh oocytes. As the study comprises relatively small numbers, our results should be interpreted with caution. Nevertheless, this is the first report documenting the health outcomes after oocyte vitrification beyond infancy. More (large-scale) follow-up studies with a prospective study set-up are needed to confirm our reassuring results and the safety of oocyte vitrification in current practice.

## Data availability

The data underlying this article are available in the article and in its online Supplementary Material.
