# Erratum to: Health of 2-year-old children born after vitrified oocyte donation in comparison with peers born after fresh oocyte donation

**DOI:** 10.1093/hropen/hoab013

**Published:** 2021-04-13

**Authors:** Marjan Van Reckem, Christophe Blockeel, Maryse Bonduelle, Andrea Buysse, Mathieu Roelants, Greta Verheyen, Herman Tournaye, Frederik Hes, Florence Belva

**Affiliations:** 1 Faculty of Medicine and Pharmacy, Vrije Universiteit Brussel, Brussels, Belgium; 2 Centre for Reproductive Medicine, Universitair Ziekenhuis Brussel (UZ Brussel), 1090 Brussels, Belgium; 3 Department of Obstetrics and Gynaecology, School of Medicine, University of Zagreb, Zagreb 10000, Croatia; 4 Centre for Medical Genetics, Universitair Ziekenhuis Brussel (UZ Brussel), 1090 Brussels, Belgium; 5 Department of Public Health and Primary Care, Environment and Health/Youth Health Care, 3000 Leuven, Belgium; 6 Department of Obstetrics, Gynecology, Perinatology and Reproduction, Institute of Professional Education, Sechenov First Moscow State Medical University of the Ministry of Health of the Russian Federation (Sechenov University), Moscow 119992, Russia

*Human Reproduction Open*, 2021, hoab002, https://doi.org/10.1093/hropen/hoab002

In the originally published version of this manuscript, the first name/family name order of each of the authors’ names were incorrect. The author list should read: “Marjan Van Reckem, Christophe Blockeel, Maryse Bonduelle, Andrea Buysse, Mathieu Roelants, Greta Verheyen, Herman Tournaye, Frederik Hes, and Florence Belva” instead of “Van Reckem Marjan, Blockeel Christophe, Bonduelle Maryse, Buysse Andrea, Roelants Mathieu, Verheyen Greta, Tournaye Herman, Hes Frederik, and Belva Florence”. This error has now been corrected online.

